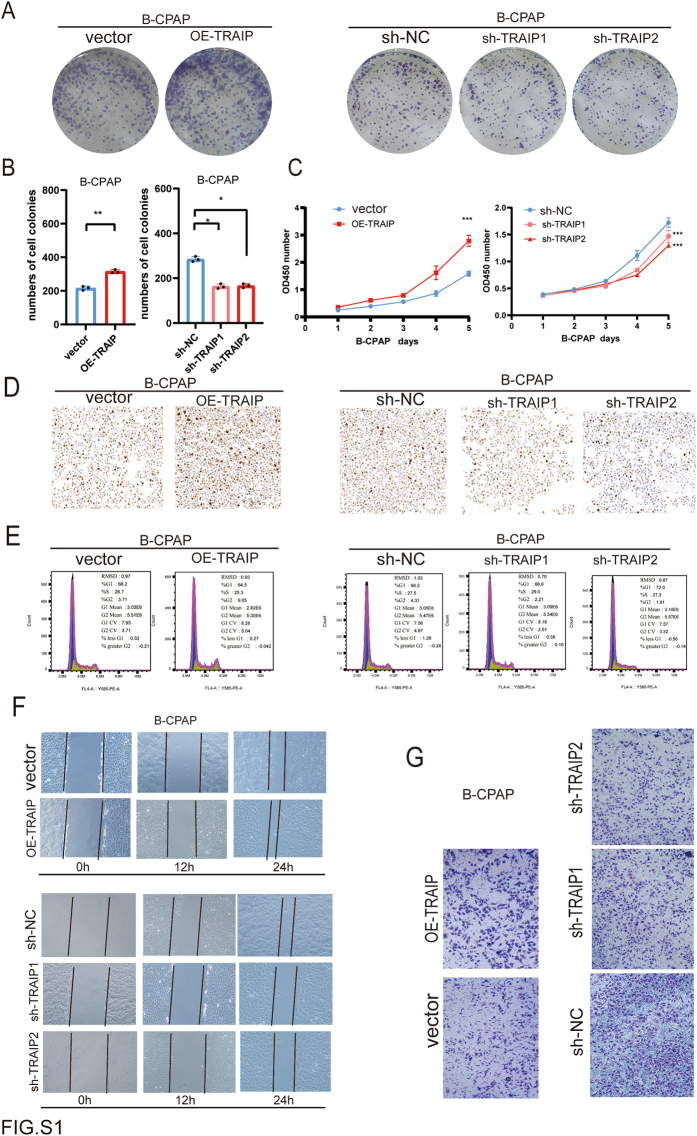# TRAIP promotes the development of papillary thyroid cancer by inhibiting TRAF2-mediated BRAF ubiquitination

**DOI:** 10.1016/j.jbc.2026.113246

**Published:** 2026-06-12

**Authors:** Huaxiao Tang, Lifang Chen, Cheng Guo, Han Wei, Hongbing Qi, Guangming Fu, Xianning Dong, Honghui Wang, Chengqin Wang

**Affiliations:** 1 Department of Pathology, The Affiliated Hospital of Qingdao University, Qingdao, Shandong, China; 2 Department of Pathology, The Affiliated Weihai Second Municipal Hospital of Qingdao University, Weihai, China; 3 Department of Pathology, School of Basic Medicine, Qingdao University, Qingdao, Shandong, China

**Keywords:** TRAF-interacting protein, papillary thyroid carcinoma, TNF receptor, BRAF, ubiquitination

## Abstract

The annual incidence of papillary thyroid cancer (PTC) has shown a steady increase in the number of cases. Advances in minimally invasive surgical techniques and the growing frequency of late-stage diagnoses have collectively heightened the complexity of PTC management and treatment. Tumor necrosis factor receptor-associated factor-interacting protein (TRAIP), which has been implicated in the progression of various malignancies, remains inadequately characterized in terms of its expression patterns and functional roles in papillary thyroid carcinoma. In this study, we implemented a comprehensive experimental strategy integrating tissue-based analyses, cellular functional assays, and *in vivo* animal models. We constructed a protein–protein interaction network and established stable cell lines with TRAIP overexpression, knockdown, knockout, and mutation. These models were subsequently analyzed using Western blotting and co-immunoprecipitation (co-IP) assays. Experimental results demonstrated significantly upregulated TRAIP expression in PTC. Silencing TRAIP markedly inhibited PTC cell proliferation and migratory potential. BRAF, a critical oncogene in PTC pathogenesis, exhibited a positive correlation with TRAIP expression both *in vitro* and *in vivo*. TRAIP was shown to regulate BRAF ubiquitination and was linked to MAPK pathway activation. However, mutation-based studies indicated that BRAF degradation did not occur *via* TRAIP-dependent ubiquitination. Furthermore, TRAIP was found to physically interact with both TRAF2 and BRAF, with TRAF2 displaying a higher binding affinity for TRAIP than BRAF. Overexpression of TRAIP reduced the interaction between TRAF2 and BRAF, whereas TRAF2 independently mediated BRAF ubiquitination. These findings demonstrate that TRAIP attenuates TRAF2-mediated BRAF ubiquitination, thereby promoting MAPK pathway activation in PTC cells, which subsequently enhances their proliferation and migration.

Within the endocrine system, thyroid cancer (TC) is one of the malignancies with a relatively high incidence rate (1). Since the 1980s, there has been a steady increase in the number of TC patients (2). Clinicopathologically, PTC is the most common type of TC, making up nearly 90% of all cases, and it typically has an excellent prognosis (3). Most PTC patients normally have satisfactory clinical outcomes. However, a subset of thyroid cancers, including invasive tumors and distant metastases to lungs and bones, exhibit aggressive biological behaviors, resulting in a dismal prognosis (4). Although multiple studies have identified several genes associated with PTC progression, the underlying pathogenesis of PTC remains incompletely understood.

TNF receptor-associated factors (TRAFs) are a family of intracellular adaptor proteins involved in signaling downstream of tumor necrosis factor (TNF) receptors and serve as key signal transduction molecules (5). Seven TRAF family members have been identified in mammals: TRAF1–TRAF7 (6). TRAF2 is the most extensively studied member of the TRAF family and participates in a wide range of cellular processes associated with cancer. (7). TRAF-interacting protein (TRAIP) was identified as a RING-type E3 ubiquitin ligase, playing a key role in inflammation, antiviral processes and apoptosis (8). Moreover, numerous studies have demonstrated that TRAIP participates in tumorigenesis, various diseases, and multiple other cellular processes. Moreover, numerous studies have demonstrated that TRAIP participates in tumorigenesis, various diseases, and multiple other cellular processes (9). It was discovered that the TRAF2 function is regulated by the interaction between the coiled-coil domain (TRAF-N) of TRAIP and the coiled-coil domain of TRAF2 (10). Bhat demonstrated that the RING domain of TRAIP, which mediates higher-order assembly, is essential for interaction with the TRAF-N domain of TRAF2, whereas the coiled-coil domain of TRAIP fails to form a complex with the TRAF-N domain of TRAF2 *in vitro* (11). Therefore, TRAIP was considered an important and suitable target for therapeutic interventions. However, the expression and function of TRAIP have not been reported in PTC.

The RAF gene family is a group of genes encoding serine/threonine protein kinases and includes three members, ARAF, BRAF, and CRAF. Mutations in the proto-oncogene BRAF is a major genetic driver in various cancers, including malignant melanoma, thyroid cancer and ovarian cancer. (12). BRAF represents the most frequently identified molecular target in TC (13, 14), and V600E represents the most prevalent BRAF mutation, situated within exons 11 and 15 of the CR3 kinase structural domain (15, 16). Therefore, regulating BRAF expression may aid in clinical diagnosis and treatment of thyroid cancer. To date, whether TRAIP can affect the expression level of BRAF and the relationship between them have not been reported.

## Results

### TRAIP protein was overexpressed and correlated with the recurrence-free survival of patients in PTC

**Figure 1:**
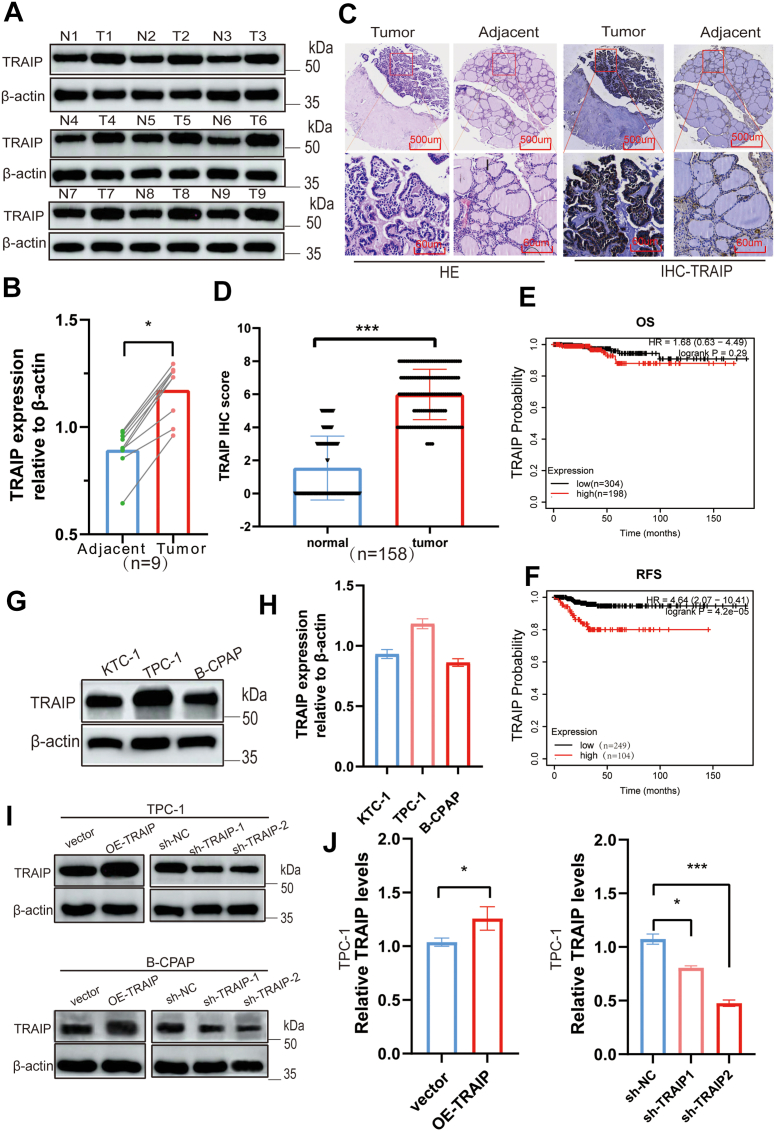
**TRAIP exhibits significantly elevated expression levels in papillary thyroid carcinoma tissues.***A* and *B*, expression of TRAIP protein in PTC and adjacent normal tissues. T: tumor, N: adjacent normal tissues. *C*, HE and IHC staining of PTC and normal tissues adjacent to tumors. revealed significantly elevated expression levels of TRAIP in PTC. *D*, scatter bar graph of TRAIP expression in tumors and paired tissues from 88 patients. *E* and *F*, Kaplan‒Meier survival curves depicting 502 papillary thyroid carcinoma patients stratified by high and low TRAIP expression levels. *G* and *H*, TRAIP protein levels in TPC-1, B-CPAP and KTC-1 cells. *I* and *J*, Western blot analysis of TRAIP silencing and overexpression efficiency. (∗∗∗*p* < 0.001, ∗∗*p* < 0.01, ∗*p* < 0.05).

By Western blotting analysis of nine paired PTC and adjacent normal tissues, we found that the expression level of TRAIP protein in tumor tissues was significantly higher than that in paired normal tissues. (Fig. 1, *A* and *B* *p* < 0.05). To further evaluate TRAIP protein levels in an independent patient cohort, we analyzed TRAIP protein levels in 158 PTC samples *via* TMAs *via* IHC. The IHC results showed the same trend as the WB findings for TRAIP detection (Fig. 1, *C* and *D p* < 0.01). To investigate the impact of TRAIP on patient survival, Survival analysis outcomes were derived from the Kaplan–Meier Plotter database, wherein the upper quartile of gene expression distribution was rigorously applied as the threshold to stratify samples into high-expression and low-expression cohorts; survival curves were subsequently generated automatically using the database integrated analytical framework (https://kmplot.com).

A study involving 502 patients with PTC revealed that high TRAIP expression was significantly associated with poorer recurrence-free survival (RFS; *HR = 4.64*) and also showed a trend toward shorter overall survival (OS; *HR = 1.68*; Fig. 1, *E* and *F*).

### TRAIP promoted malignant proliferation of thyroid cancer cells both *in vitro* and *in vivo*

**Figure 2:**
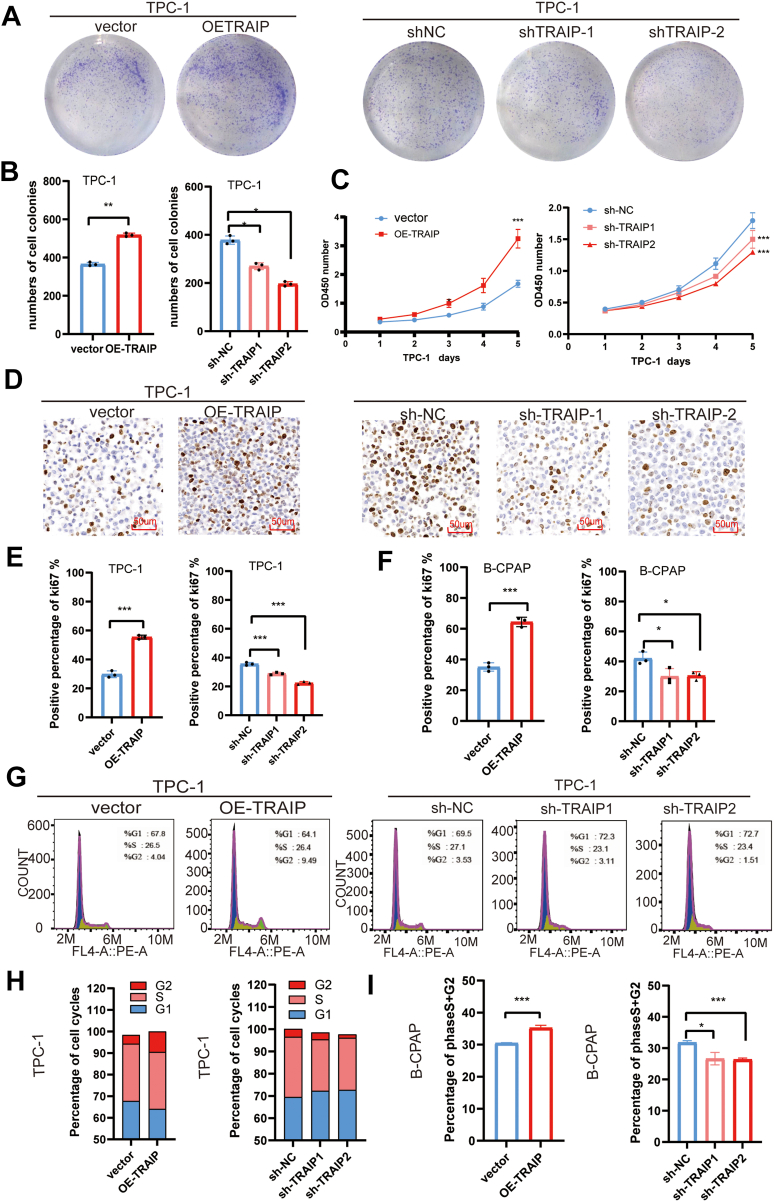
**TRAIP facilitates the proliferation and migration of thyroid cancer cells.***A* and *B*, cell cloning experiment image, Histograms showing the number of colonies. *C*, cell proliferation was detected by a CCK-8 assay. *D*–*F*, immunohistochemical analysis of Ki67 expression in cells in the TRAIP-knockdown and TRAIP-overexpressing groups; histograms showing the percentage of Ki67-positive cells. *G*–*I*, flow cytometry showing the cell cycle distribution of the cell lines (∗∗∗*p* < 0.001, ∗∗*p* < 0.01, ∗*p* < 0.05).

To investigate the role of TRAIP in thyroid cancer cells, we examined TRAIP protein levels in the TPC-1, B-CPAP and KTC-1 cell lines *via* western blotting (Fig. 1, *G* and *H*). We subsequently selected TPC-1 and B-CPAP cells for TRAIP silencing and overexpression experiments (Fig. 1, *I* and *J*). The results of the colony formation assay revealed that cell proliferation was attenuated after TRAIP knockdown, whereas the proliferation of TRAIP-overexpressing cells was promoted (*p < 0.05*) (Figs. 2, *A* and *B*, and S1, *A* and *B*). and the results of the CCK-8 assays yielded the same trends (*p < 0.05*) (Figs. 2*C*, and S1*C*). IHC of Ki67 in the OETRAIP group likewise demonstrated elevated proliferative activity, whereas the percentage of Ki67 in the sh-TRAIP group was significantly lower compared to the control group. (*p < 0.05*) (Figs. 2, *D*–*F*, and S1*D*). In the flow cytometry results, the proportion of cells in the S and G2 phases in the OETRAIP group was significantly higher than that in the control group, whereas the opposite result was observed in the sh-TRAIP group (*p < 0.05*) (Figs. 2, *G*–*I*, and S1*E*).

**Figure 3:**
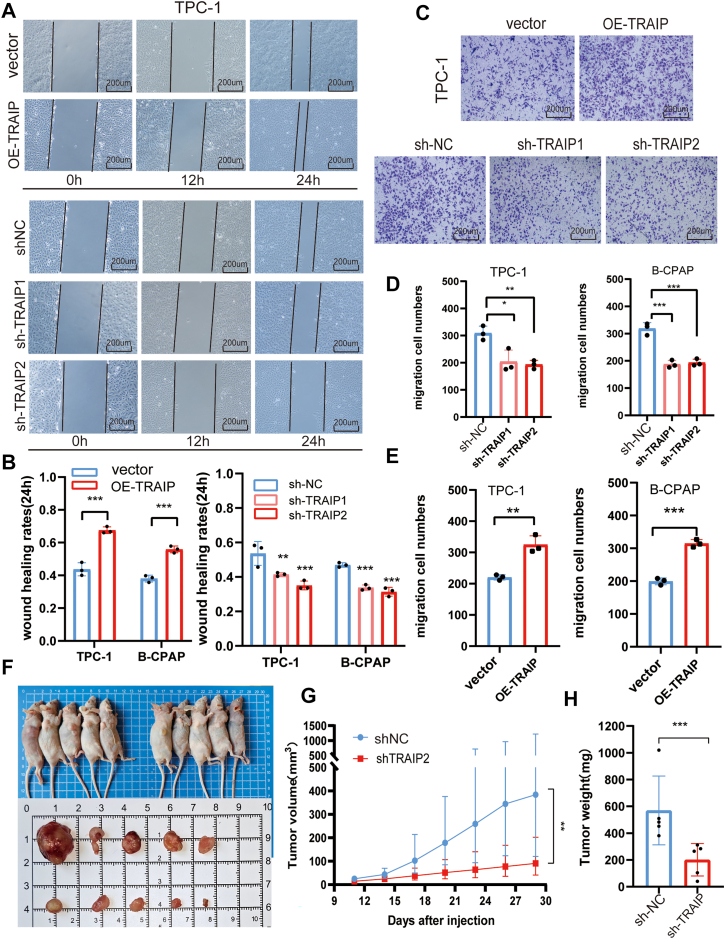
**TRAIP facilitates the proliferation and migration of thyroid cancer cells.***A* and *B*, wound-healing assays were used to determine the effects of TRAIP knockdown or overexpression on cell migration. *C*–*E*, trans well assay showing the number of cells migrating in the TRAIP gene knockdown or overexpression group. *F*, Sh-Ctrl and sh-TRAIP TPC-1 cells were injected under the skin of nude mice. *G* and *H*, statistical graphs of the weight and volume of the subcutaneous tumors were generated, and the formula for tumor volume measurement was as follows: volume (mm3) = (width2 × length)/(*∗∗∗p* < 0.001, ∗∗*p* < 0.01, *∗p* < 0.05).

We assessed the migration ability of PTC cells *via* wound healing and trans well assays. Notably, TRAIP overexpression accelerated horizontal migration in the OE-TRAIP group, whereas TRAIP silencing had the opposite effect (*p < 0.05*) (Figs. 3, *A* and *B*, and S1*F*). The expression level of TRAIP was positively correlated with the vertical migration capacity of cells (*p < 0.05*) (Figs. 3, *C*–*E*, and S1*G*). To further investigate the functional role of TRAIP *in vivo*, we established a xenograft nude mouse model by subcutaneously inoculating TPC-1 cells carrying shTRAIP2 and negative control cells into nude mice. The subcutaneous tumors were eventually excised after 30 days (Fig. 3*F*). The volume and weight of tumors induced in nude mice inoculated with TRAIP-knockdown cells were both reduced compared to the control group(*p < 0.05*) (Fig. 3, *G* and *H*). Silencing TRAIP clearly inhibited the growth of tumors in xenograft nude mice. All experimental results collectively demonstrated that TRAIP facilitates the malignant proliferation of PTC cells both *in vitro* and *in vivo*.

### TRAIP protein expression is correlated with BRAF, but the TRAIP gene does not regulate BRAF gene expression

**Figure 4:**
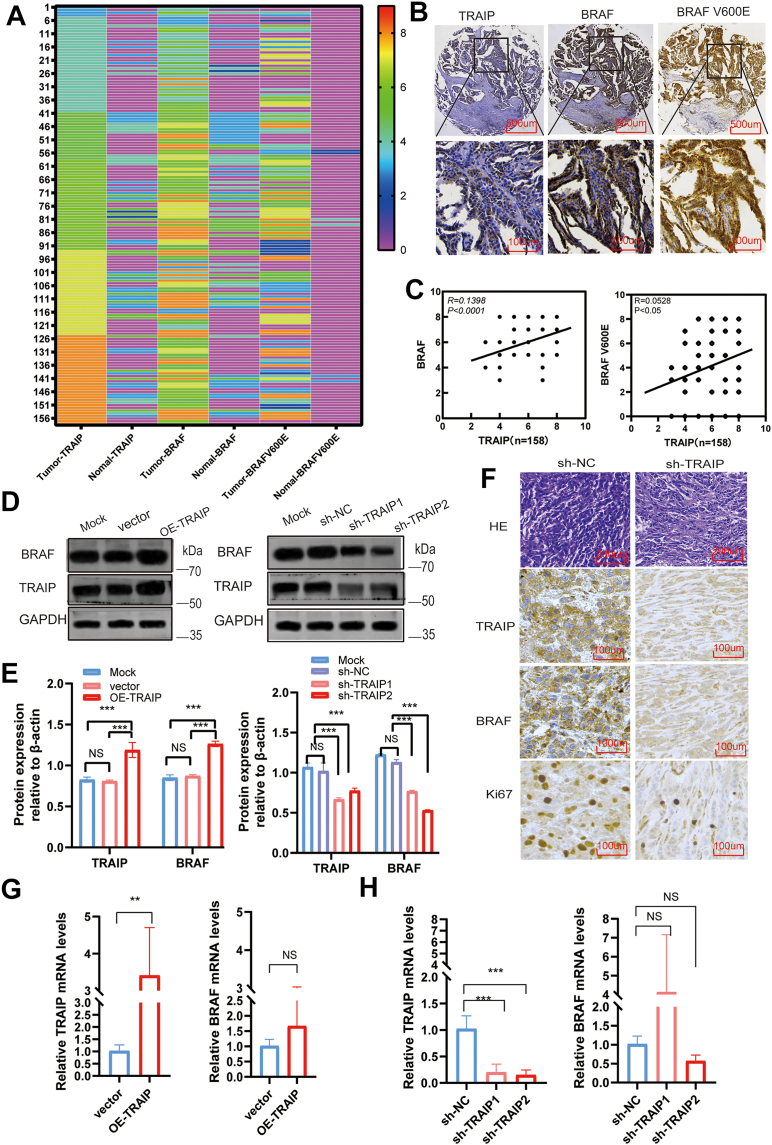
**TRAIP demonstrates a statistically significant correlation in protein expression levels with BRAF.***A*, heatmap of the TRAIP and BRAF immunohistochemical staining scores of 158 PTC tissues and paracancerous tissues. *B*, immunohistochemical overexpression of TRAIP and BRAF, BRAFV600E in the same patient. *C*, dot plot of the correlation between TRAIP and BRAF, BRAFV600E. *D* and *E*, Western blot analysis of the protein expression of TRAIP and BRAF in TRAIP-knockdown and TRAIP-overexpressing cells. *F*, HE-stained images of tumors in the vector and shTRAIP groups and immunohistochemical images of TRAIP, BRAF, and Ki67. *G* and *H*, PCR detection of the mRNA expression of TRAIP and BRAF in TRAIP-knockdown and TRAIP-overexpressing cells.

The MAPK pathway is a classical pathway in papillary thyroid cancer. BRAF, whose V600E mutation plays an important role in the development of thyroid cancer, is a classical protein in this pathway. To investigate whether TRAIP and BRAF expression are correlated in PTC, we performed simultaneous immunohistochemical staining for BRAF and BRAFV600E. TRAIP, BRAF, and BRAF V600E all showed high expression levels in tumor tissues (Fig. 4*A*). High TRAIP expressions in tumor tissues were frequently accompanied by high expressions of both BRAF and BRAF V600E (Fig. 4*B*). Statistical analysis of TRAIP and BRAF IHC scores in tumors showed a positive correlation between TRAIP with BRAF and BRAF V600E(*p < 0.05*) (Fig. 4*C*). To further explore the relationship between TRAIP and BRAF, proteins were extracted from cells stably transduced with lentivirus to perform WB experiments. Sh-TRAIP reduced BRAF protein levels, whereas TRAIP overexpression increased BRAF protein levels. (Fig. 4, *D* and *E*). IHC analysis of tumors in nude mice showed lower levels of BRAF and Ki-67 in the sh-TRAIP group than in the vector group (Fig. 4*F*). To determine whether a direct transcriptional or post-transcriptional regulatory relationship exists between TRAIP and BRAF at the gene expression level, we assessed BRAF mRNA abundance following both TRAIP overexpression and knockdown, and observed no significant changes (Fig. 4, *G* and *H*).

### TRAIP interacts with BRAF and activates the MAPK signaling pathway

**Figure 5:**
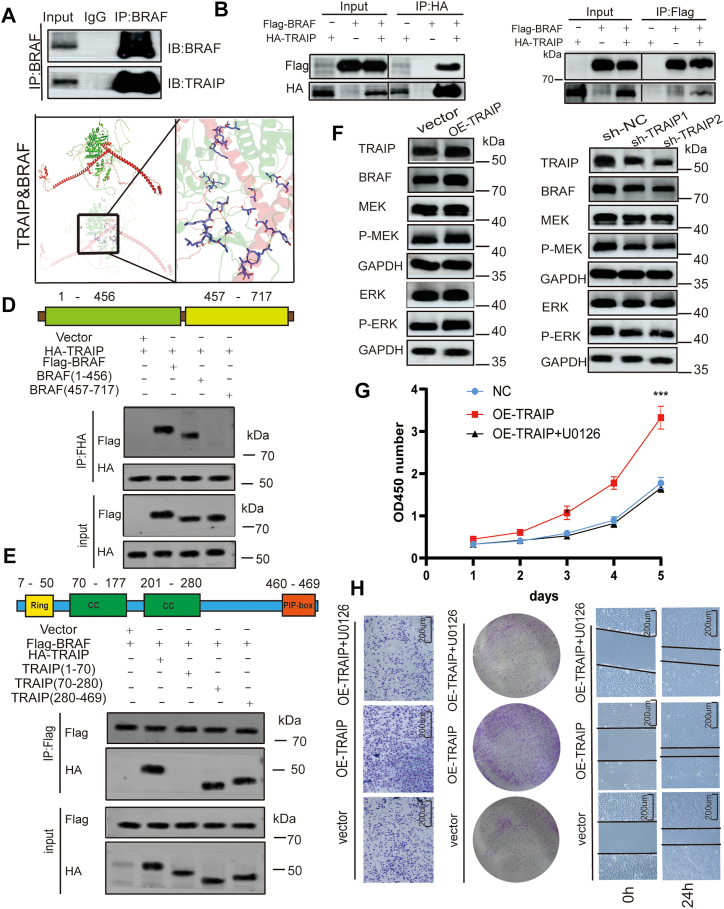
**TRAIP physically interacts with BRAF and modulates MAPK pathway activation.***A*, co-IP detection of endogenous interactions between TRAIP and BRAF. *B*, co-IP detection of exogenous interactions between TRAIP and BRAF. *C*, protein docking modeling of TRAIP and BRAF. *D* and *E*, co-IP analysis of truncated TRAIP interaction with BRAF. *F*, overexpressing or knocking down TRAIP affects total BRAF protein levels and subsequently affects downstream signaling. *G* and *H*, the proliferation and migration abilities of TRAIP overexpressing cells were weakened after treatment with the MEK inhibitor (U0126, 10 umol/L).

**Figure 6:**
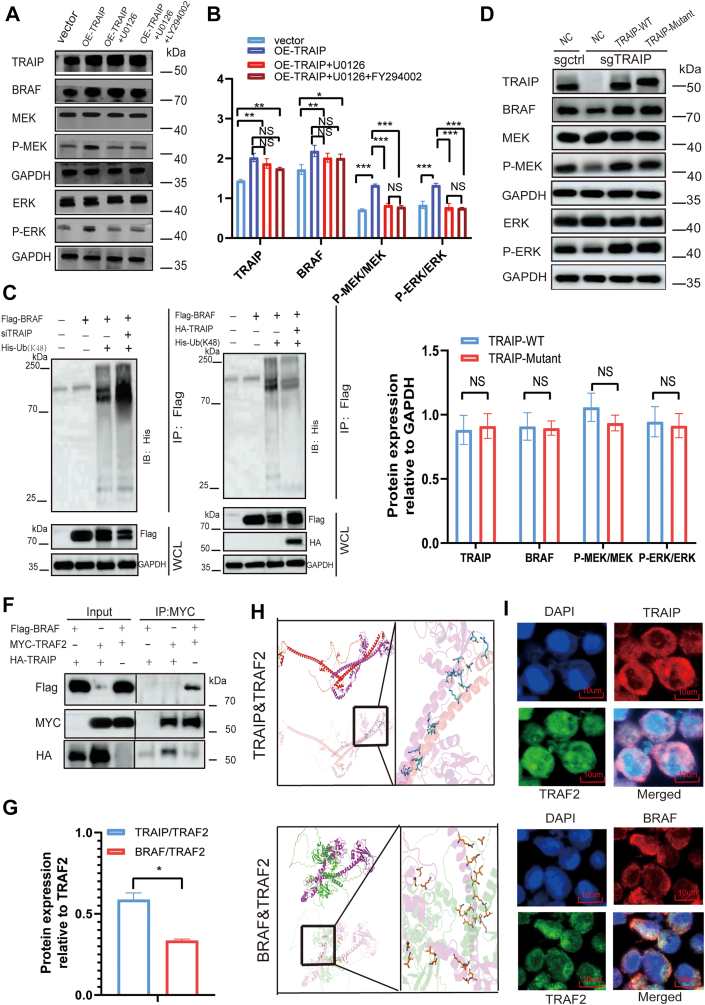
**The interaction among TRAIP, TRAF2, and BRAF modulates the ubiquitination modification of BRAF.***A* and *B*, the expression levels of key MAPK pathway proteins were assessed following treatment with the selective PI3K inhibitor LY294002. *C*, knockdown or overexpression of TRAIP affected BRAF ubiquitination. *D* and *E*, Western Blot analysis of MAPK pathway in TPC-1 cells transfected with TRAIP wild type and enzyme-dead mutant overexpression plasmid after TRAIP knockout. *F* and *G*, co-IP detection of exogenous interactions between TRAF2, TRAIP and BRAF. *H*, protein docking modeling of TRAIP and TRAF2, TRAF2 and BRAF. *I*, immunofluorescence analysis further demonstrated the co-localization of TRAIP with TRAF2 and TRAF2 with BRAF.

Above findings suggest that TRAIP expression affects BRAF expression in PTC. To explore the relationship between TRAIP and BRAF, we investigated the interaction between endogenous BRAF and TRAIP. The results demonstrated that BRAF is a protein-binding partner of TRAIP (Fig. 5*A*). We then transfected the HA-TRAIP and Flag-BRAF plasmids into TPC-1 cells and extracted the proteins for exogenous interaction assays after 48 h, which further confirmed that TRAIP can bind to BRAF (Fig. 5*B*). Finally, we performed rigid protein–protein docking between TRAIP and BRAF, which also revealed that TRAIP can stably interact with BRAF(Binding energy: -14.5 kcal/mol) (Fig. 5*C*, Table S1). To precisely map the direct protein–protein interaction interface between TRAIP and BRAF, we generated a series of domain-specific mutants and conducted co-immunoprecipitation (co-IP) assays. These experiments demonstrated that TRAIP selectively associates with the non-kinase region of BRAF, with primary binding occurring at coiled-coil (CC) domain and C-terminal region of TRAIP (Fig. 5, *D* and *E*). Interestingly, our results reveal that knockdown of TRAIP in TPC-1 cells reduces BRAF expression, thereby inhibiting downstream phospho-MEK (S218 + S222) and phospho-ERK (T202 + Y204). Conversely, the overexpression of TRAIP increased the levels of phospho-MEK (S218 + S222) and phospho-ERK (T202 + Y204) (Fig. 5*F*). Furthermore, upon treatment of OETRAIP cells with the MEK inhibitor (U0126), a reduction was observed in both cellular proliferation and migration capacities (Fig. 5, *G* and *H*). To eliminate potential cross-talk from the PI3K/Akt signaling pathway, we conducted a combinatorial inhibition experiment using the specific PI3K inhibitor LY294002 and subsequently evaluated the expression levels of key MAPK pathway proteins. These results demonstrate that TRAIP-mediated regulation of papillary thyroid carcinoma (PTC) occurs independently of the PI3K/Akt pathway (Fig. 6, *A* and *B*).

### TRAIP influences ubiquitination modification of BRAF but does not depend on its enzyme activity

To clarify the regulatory mechanism underlying TRAIP–BRAF interactions, we performed an exogenous ubiquitination assay using a K48-linked polyubiquitin-specific antibody and found that BRAF ubiquitination levels increased upon TRAIP knockdown, whereas they decreased upon TRAIP overexpression (Fig. 6*C*). These findings indicate that TRAIP regulates the ubiquitination of BRAF in thyroid cancer. We restored wild-type (WT) TRAIP and the catalytically inactive mutant TRAIP (C7/10A) in TRAIP-knockout TPC-1 cells and found that neither the WT nor the enzyme-dead variant induced significant changes in the activation of the MAPK signaling pathway (Fig. 6, *D* and *E*) (17, 18). The experimental results indicate that TRAIP regulates the ubiquitination of BRAF independently of its E3 ligase activity.

### TRAIP competitively binds to TRAF2, leading to a reduction in TRAF2-mediated ubiquitination modification of BRAF

**Figure 7:**
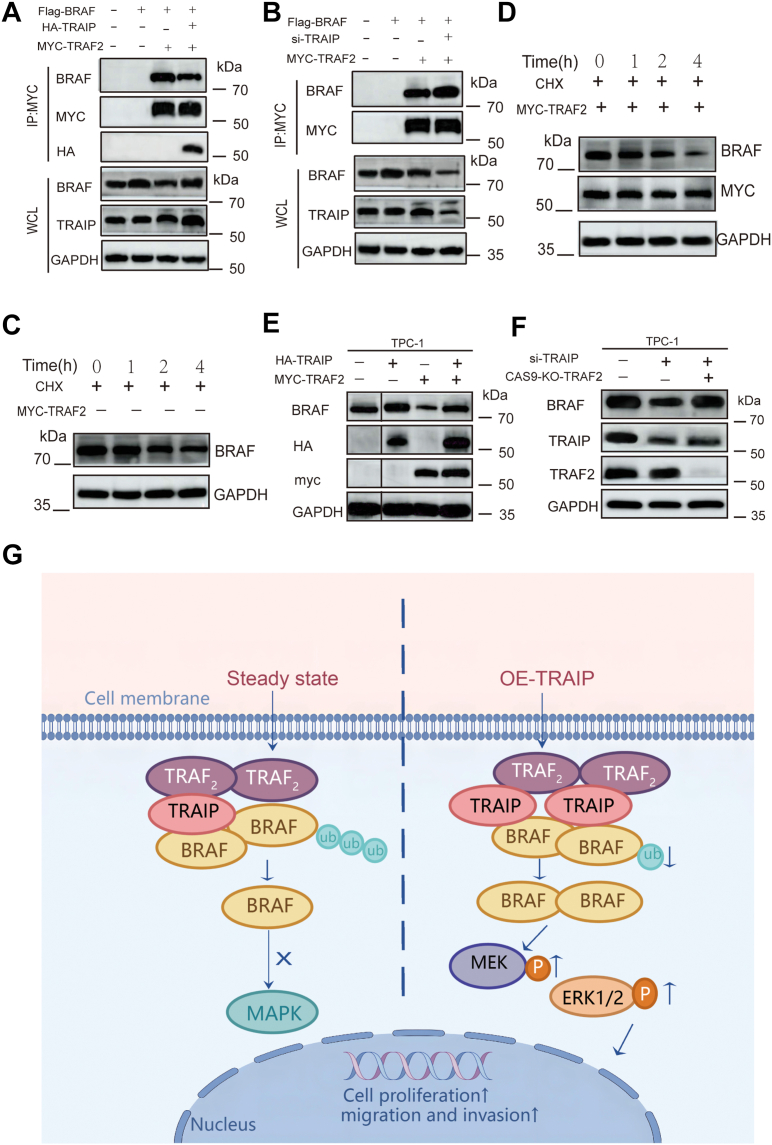
**TRAIP regulates BRAF expression through competitive binding mechanisms.***A* and *B*, TRAIP knockdown resulted in an increased interaction between TRAF2 and BRAF. Consistently, overexpression of TRAIP inhibited the interaction between TRAF2 and BRAF. *C* and *D*, CHX-treated cells exhibit accelerated ubiquitination of BRAF after TRAF2 overexpression. *E*, BRAF expression was reduced after overexpression of TRAF2 but increased after simultaneous overexpression of TRAIP. *F*, BRAF expression was reduced after TRAIP knockdown but restored if TRAF2 was knocked out at the same time. *G*, TRAIP induced functional inhibition of TRAF2 by binding directly to TRAF2 and BRAF, reducing the level of TRAF2-mediated BRAF ubiquitination and activating the MAPK pathway.

To further explore the underlying mechanisms through which TRAIP regulates BRAF activity. Prior research has indicated a close interaction between TRAF2 and TRAIP, and additional studies have shown that TRAF2 can bind to BRAF and mediate its ubiquitination (11, 19). To further verify this, Co-IP assays confirmed that TRAF2 can bind to TRAIP and BRAF (Fig. 6*F*). Quantitative data demonstrate that TRAF2 exhibits a higher binding affinity for TRAIP protein compared to BRAF (Fig. 6*G*). We also attempted to construct protein docking models for TRAIP-TRAF2 and TRAF2-BRAF, which confirmed that TRAF2 can directly bind to both TRAIP and BRAF. Furthermore, in comparison to TRAF2-BRAF (Binding energy: -23.4 kcal/mol), the TRAIP-TRAF2(Binding energy:-36.4 kcal/mol) complex demonstrates enhanced binding energy (Fig. 6*H*, Table S1). Immunofluorescence analysis further demonstrated the co-localization of TRAIP with TRAF2, as well as that of TRAF2 with BRAF (Fig. 6*I*). Based on this information, we speculate that the interaction between TRAIP and TRAF2 interferes with the binding of TRAF2 to BRAF. To further investigate the binding relationship among the three, we conducted IP experiments to observe the binding of TRAF2 and BRAF. Upon overexpressing TRAIP, the binding of TRAF2 and BRAF notably decreased, while the expression of BRAF in total protein increased. In contrast, when TRAIP was knocked down, the interaction of TRAF2 and BRAF was enhanced, and the protein expression level of BRAF decreased (Fig. 7, *A* and *B*). To assess the ubiquitination effect of TRAF2 on BRAF, we administered cycloheximide (CHX), a protein synthesis inhibitor, to monitor BRAF degradation. Our findings revealed that TRAF2 overexpression markedly enhanced the ubiquitination modification of BRAF (Fig. 7, *C* and *D*). To further determine whether TRAIP regulates BRAF ubiquitination through TRAF2, we examined BRAF expression under various combinations of TRAIP and TRAF2 expression levels. We found that TRAIP overexpression in cells with elevated TRAF2 led to a corresponding reduction in BRAF protein levels (Fig. 7*E*). Additionally, BRAF expression was reduced upon TRAIP knockdown but was rescued when TRAF2 was simultaneously knocked out (Fig. 7*F*), suggesting that TRAF2 acts as a critical mediator between TRAIP and BRAF. All these results demonstrate that TRAIP influences the binding between TRAF2 and BRAF, thereby attenuating TRAF2-mediated ubiquitination of BRAF, which enhances activation of the MAPK signaling pathway and promotes the progression and development of PTC (Fig. 7*G*).

## Discussion

In the general population, approximately 60% of adults with one or more nodules (20). PTC is the most prevalent type of thyroid malignancy, and its incidence has shown a continuous upward trend in recent years. and its incidence has shown a continuous upward trend in recent years (21, 22). Risk factors include radiation exposure, the presence of thyroid nodules, and genetic factors (23). Although many studies have investigated various mechanisms of thyroid cancer, including gene mutations, tumor immunity, and other related aspects, the precise pathogenic mechanisms remain incompletely understood. (24, 25, 26). Currently, numerous challenges persist in the diagnosis and treatment of PTC. More therapeutic targets need to be identified to improve treatment outcomes.

TRAIP was initially identified as a protein capable of interacting with TRAF1 and TRAF2 (27), playing a critical role in maintaining genomic integrity (28). During mitosis, TRAIP can maintain genomic stability and regulate mitotic progression (29). TRAIP is a key enzyme involved in replicon segregation during the termination phase of DNA replication and plays a regulatory role in mitotic progression (30). In the context of DNA damage repair, TRAIP regulates homologous recombination and influences the choice of interstrand crosslink repair pathways (31, 32). Multiple studies have demonstrated that TRAIP plays a role in the biological processes associated with various types of tumors (33). In hepatocellular carcinoma, TRAIP expression is negatively correlated with prognosis (34). TRAIP expression is strongly correlated with the infiltration of diverse immune cell types, including B cells, CD8+ T cells, neutrophils and dendritic cells in lung adenocarcinoma (LUAD) (35). In breast epithelial cells, TRAIP can interact with the protein tyrosine kinase Syk (36). TRAIP has oncogenic effects on triple-negative breast cancer through RB-E2F signaling and EMT (9). Studies have demonstrated that TRAIP is significantly overexpressed in prostate cancer (PCa) tissues and tumor cell lines; this overexpression enhances the proliferation, invasion, and migration capabilities of tumor cells in these models. This oncogenic effect is mediated through induction of cell cycle arrest and suppression of apoptosis—findings confirmed by multiple functional assays and by analyzing the expression levels of key cell cycle– and apoptosis-regulating proteins in cultured cells. Moreover, TRAIP interacts with TRAF2 to activate the PI3K/AKT signaling pathway (37). TRAIP promotes the proliferation, migration, and invasion of tongue squamous cell carcinoma (TSCC). Bioinformatics analysis, mass spectrometry, and co-immunoprecipitation experiments collectively suggest that DDX39A is a potential interacting partner of TRAIP. DDX39A has been validated as an oncogene in multiple tumor types and is likely to play a critical role in tumor cell proliferation and metastasis. TRAIP may regulate these cellular processes—particularly those involved in epithelial-mesenchymal transition (EMT) and the Wnt/β-catenin signaling pathway—through its interaction with DDX39A (38).Recent studies have substantiated that TRAIP exerts an oncogenic function in osteosarcoma and bladder cancer (39, 40). Our study demonstrates that TRAIP is significantly upregulated in thyroid cancer tissues. Silencing TRAIP suppresses the proliferative and migratory capacities of PTC cells, whereas its overexpression enhances these capabilities. These findings indicate that TRAIP plays a critical role in the progression of PTC. However, the molecular mechanisms governing TRAIP expression remain incompletely characterized. A prior study demonstrated that SUMOylation modulates TRAIP’s nuclear translocation and protein stability (41).Another identified miR-1246 as a direct regulator of TRAIP, proposing its potential utility as a diagnostic or prognostic biomarker in lung adenocarcinoma (42). Furthermore, in lung cancer, the long non-coding RNA SLC7A11-AS1 has been shown to upregulate TRAIP expression through sequence-specific inhibition of miR-4775 (43). The BRAF gene, which encodes the B-Raf protein, is a key member of the RAF kinase family (44, 45). As an upstream regulator of the RAS-RAF-MEK-ERK signaling pathway, BRAF participates in the sequential activation of this pathway and modulates multiple cellular processes (46, 47). BRAF gene mutations are a well-established pathogenic mechanism in differentiated PTC (48, 49). The BRAF mutation is more commonly observed in sporadic papillary thyroid carcinoma, particularly in the highly cellular variant and aggressive microcarcinomas among adult patients (50). In this study, a significant positive correlation was observed between TRAIP and BRAF protein expression levels in clinical tumor tissues.

To further investigate this relationship, we confirmed that TRAIP is correlated with BRAF through both endogenous and exogenous Co-IP experiments. Thus, a key question arises: how does TRAIP regulate the expression of BRAF? Structurally, TRAIP contains a RING domain and may function as an E3 ligase, either directly or indirectly participating in the ubiquitination of BRAF (51). However, mutating the RING domain of TRAIP in this experiment did not affect BRAF ubiquitination, indicating that BRAF ubiquitination is independent of TRAIP’s E3 ubiquitin ligase activity. To further explore the mechanism through which TRAIP influences BRAF, a comprehensive review of relevant literature was conducted. Lee *et al.* demonstrated that, *in vitro*, the RING domain of TRAIP interacts with the TRAF-N domain of TRAF2 to sequester monomeric TRAF2, thereby inhibiting the assembly of functional, active trimeric TRAF2 complexes (10). Bhat *et al.* suggested that the coiled-coil structural domain of TRAIP interacts with the TRAF2 coiled-coil structural domain to inhibit TRAF2 function (11). A previous study showed that TRAF2 can directly mediate BRAF Lys48-related ubiquitination through binding to BRAF in non-small cell lung cancer (NSCLC) (19). To achieve this objective, we performed additional experimental validation. Co-immunoprecipitation assays and molecular docking analyses jointly confirmed direct pairwise interactions among TRAIP, TRAF2, and BRAF. Furthermore, molecular docking simulations coupled with quantitative binding affinity measurements indicated that the TRAIP–TRAF2 interaction exhibits higher binding strength compared to the TRAIP–BRAF and TRAF2–BRAF interactions. Functional perturbation experiments revealed that TRAIP overexpression attenuated the TRAF2–BRAF interaction, whereas TRAIP knockdown enhanced TRAF2-mediated ubiquitination of BRAF. Notably, in TRAIP-knockout cells, BRAF protein levels were restored upon TRAF2 depletion. Collectively, these findings establish TRAF2 as a critical intermediary through which TRAIP modulates BRAF expression. Based on our findings, future clinical trials may evaluate TRAIP inhibitors either as monotherapy or in combination with BRAF inhibitors for the treatment of PTC. Furthermore, targeted therapeutic strategies aimed at modulating TRAIP and/or TRAF2 through specific binding site intervention hold promise for enhancing therapeutic efficacy in PTC and improving patient quality of life.

In summary, TRAIP plays a crucial role in PTC and exhibits a positive correlation with BRAF. By competitively interfering with the binding of TRAF2 and BRAF, it prevents the ubiquitination modification of BRAF by TRAF2, thereby stabilizing BRAF expression. This subsequently activates the phosphorylation of MEK and ERK, promoting the progression and development of PTC (Fig. 7*H*). Collectively, these findings indicate that therapeutic strategies targeting the inhibition of TRAIP function could serve as promising approaches for both the prevention and treatment of PTC.

Although this study provides an initial characterization of TRAIP’s involvement in the initiation and progression of PTC and partially delineates its underlying molecular mechanisms, several methodological and conceptual limitations warrant acknowledgment. First, the clinical data and tissue samples used in this study were obtained solely from a single tertiary medical center and lacked longitudinal follow-up information, which may introduce selection bias and limit the external validity of the findings. Second, while the study comprehensively characterized the downstream TRAIP–TRAF2 interaction and its functional impact on MAPK signaling, the upstream regulatory mechanisms governing TRAIP expression remain uncharacterized, resulting in an incomplete understanding of the broader TRAIP-centered regulatory network. Third, the structural determinants and precise molecular interfaces that mediate the ternary interactions among TRAIP, TRAF2, and BRAF have not been systematically elucidated or experimentally validated, hindering the development of a high-resolution mechanistic model essential for structure-guided therapeutic intervention. Finally, the study lacks a systematic evaluation of translational potential, thereby limiting definitive conclusions about its immediate clinical applicability. Systematically addressing these methodological and conceptual limitations constitutes a primary objective of our ongoing and future research initiatives.

## Experimental procedures

### Cell lines and tissues

The human thyroid cancer cell lines TPC-1, KTC-1 and B-CPAP were purchased from Starfish Biological Cell Bank. In a humidified incubator at 37 °C with 5% CO_2_, cells were incubated in DMEM supplemented with 10% fetal bovine serum and 1% penicillin/streptomycin. A total of 158 tissue samples for this study were collected from the Weihai Municipal Second Hospital of Qingdao University. All pathological diagnoses were independently reviewed and confirmed by two pathologists, and low-risk tumors such as noninvasive follicular thyroid neoplasm with papillary-like nuclear features(NIFTP) were excluded. None of the enrolled patients received neoadjuvant chemotherapy or radiotherapy prior to surgical intervention. This work was conducted according to the guidelines of the Declaration of Helsinki and approved by the Ethics Committee of The Affiliated Hospital of Qingdao University(WHFY-YXLLWYH-L2025019).

### Immunohistochemistry

Anti-TRAIP (Abcam, dilution 1:100) and anti-BRAF (Abcam, dilution 1:100) antibodies were employed for immunohistochemical staining following standard protocols as specified by the manufacturer. The immunohistochemical results were evaluated by the sum of staining intensity and positive cell proportion scores (range 0–8). The staining intensity score was based on: 0 (no staining), 1 (weak), 2 (moderate), 3 (strong). The positive cell proportion score was based on: 0 (no staining), 1 (<1/100), 2 (1/100–1/10), 3 (1/10–1/3), 4 (1/3–2/3), 5 (>two-thirds).

### Vector and plasmid construction

LV3-hsa-TRAIP-318 (sh-TRAI), LV3-hsa-TRAIP-543 (sh-TRAIP), and LV5-hsa-TRAIP-homo (OE-TRAIP) were constructed using lentiviral vectors (GenePharma, Shanghai, China). Empty lentiviral vectors LV3 (sh-Ctrl) or LV5 (NC) were used as negative controls. The HA-TRAIP, Flag-BRAF, and myc-TRAF2 plasmids were bought from GeneChem in Shanghai, China. All plasmids were constructed using standard procedures and their integrity was confirmed by sequencing.

### Cell transfections

TPC-1 and B-CPAP cells were transduced with corresponding lentiviral vectors to silence or overexpress TRAIP, using empty vector lentiviruses as negative controls. Puromycin was used to select stably transduced cells after transfection. Lipofectamine 3000 reagent (L3000001, Invitrogen) was used for plasmid transfection. Single-plasmid and multiple-plasmid transfection were performed according to the experimental design scheme, and the protein was extracted 48 h after transfection.

### Real-time PCR analysis

Total mRNA was isolated from cells and fresh surgical specimens using Trizol reagent. Using the Applied Biosystems QuantStudio three sequence detection system, quantitative real-time PCR was carried out. To eliminate the impact of variations in RNA loading and reverse transcription efficiency, gene expression levels were normalized to GAPDH. All primers were designed using Primer 5.0 software and experimentally validated for specificity.

### Western blot analysis

Protein samples were denatured and quantified, separated by 10% SDS-polyacrylamide gel electrophoresis, and then transferred onto polyvinylidene difluoride membranes. After blocking and incubation with primary antibodies, the membranes were incubated with horseradish peroxidase-conjugated secondary antibodies. Finally, proteins were detected using enhanced chemiluminescence (ECL) reagents. The antibodies employed included anti-TRAIP (ProteinTech, 1:1000 dilution) and anti-BRAF (Abcam, 1:2000 dilution). Protein expression levels were quantified *via* densitometric analysis using ImageJ software, and subsequent statistical analyses along with bar graph generation, were conducted using GraphPad Prism 9.

### Cell proliferation analysis

Cells from each group were seeded into 96-well plates with approximately 2000 cells per well, followed by incubation with 10 μl of CCK-8 reagent (Dojindo, Shanghai, China) for 2 h. Data were recorded on days 1 to 5. According to the set parameters for the colony formation assay, cells were seeded into 6-well plates and cultured for approximately 10 days. Following that, methanol was used to fix the cells, and crystal violet was used for staining. Lastly, the imaging software was used to count the number of cells.

### Transwell assays

Add medium with serum to the lower transwell chamber, and add serum-free medium containing transduced cells to the upper chamber. Take 24 h for incubation, and then use 4% paraformaldehyde to remove all of the medium and fix the cells for 4 hours. After fixation, stain the cells with 0.1% crystal violet.

*Wound healing assay*. Wounds were produced when cells reached 90% confluence. 0 and 24 h microscope was used to take pictures of the wounds. Using ImageJ, all of the photographs were examined. The value was calculated using the formula: (area at 0 h - area at 24 h)/area at 0 h × 100%.

### Cell cycle analysis of flow cytometry (FCM)

Using trypsin without EDTA, the cells were gathered in six-well plates. After centrifugation, cells were fixed with 70% ethanol and stored overnight at 4 °C. Then RNaseA (20 μg/ml) and propidium iodide (50 μg/ml) were added, followed by incubation for 30 min in the dark. Then,a flow cytometer was used to examine the stained cells.

### Xenograft assays in nude mice

Female BALB/c nude mice were divided into two groups of five mice each and subcutaneously injected with sh-NC or sh-TRAIP2 TPC-1 cells at 5 weeks of age. A volume of 0.1 ml of cell suspension (5 × 10^6^ cells/ml), prepared in phosphate-buffered saline (PBS), was administered *via* intradermal injection into the lateral right axillary region of athymic nude mice. Tumor dimensions were monitored and recorded every 3 days using standardized caliper measurements. The predefined humane endpoint was defined as the emergence of unequivocal clinical signs of distress, including but not limited to lethargy, weight loss exceeding 20%, impaired mobility, or labored respiration. Mice were humanely euthanized by CO_2_ inhalation before reaching this endpoint, strictly in accordance with institutional animal care and use guidelines. This study was approved by Experimental Animals Ethics Committee of Qingdao University(20231209BALB/c-Nude1020240201063).

### Coimmunoprecipitation (co-IP) assays

Co-IP assays were performed using the Biolinkedin Protein A/G Immunoprecipitation Kit (IK-1004) according to the manufacturer's instructions. IgG antibody was used as a control, and the target antibody was added to the samples for immunoprecipitation, followed by overnight incubation at 4 °C. Following the addition of magnetic beads to each sample, the mixtures were incubated for 2 h. The beads were washed, resuspended in loading buffer, and heated to denature the proteins for separation from the beads.

### Ubiquitination assay

TPC-1 cells were treated with CHX and/or MG132 for 0 to 4 h before harvest, 48 h after transfection. Thereafter, total proteins were extracted and analyzed by immunoblotting.

### Molecular docking analysis

Protein structures were obtained from the Protein Data Bank (http://www.rcsb.org/). Rigid-body protein-protein docking was performed using GRAMM-X (http://gramm.compbio.ku.edu/) to investigate molecular interactions between proteins. Protein-protein interactions were visualized and analyzed using PDBePISA (https://www.ebi.ac.uk/pdbe/pisa/) and PyMOL (version 2.4).

### Statistical analyses

The mean standard deviation(SD)of all the data is used to represent them, and at least three separate runs of each experiment were conducted separately. Statistical analyses were conducted with GraphPad Prism 8.0 software, utilizing Student's *t* test and Two-way ANOVE analyses to assess significance. Statistical significance was represented as ∗*p* < 0.05; ∗∗*p* < 0.01; ∗∗∗*p* < 0.001.

## Data availability

All data are contained within the manuscript.

## Supporting information

This article contains supporting information.

## Conflict of interest

The authors declare that they have no conflicts of interest with the contents of this article.

## Figures and Tables

**Figure S1:**