# Supplementary material for: TRAIP promotes the development of papillary thyroid cancer by inhibiting TRAF2-mediated BRAF ubiquitination
Source: J Biol Chem. 2026 Jun 12;302(8):113246. doi: 10.1016/j.jbc.2026.113246 (PMC13355724; doi:10.1016/j.jbc.2026.113246)
Supplement: Figure S1 legend [file mmc1.docx]

Figure S1 （A,B）Cell cloning experiment image，Histograms showing the number of colonies. （C）Cell proliferation was detected by a CCK-8 assay at 1, 2, 3, 4, and 5 d. （D）Immunohistochemical analysis of Ki67 expression in cells in the TRAIP-knockdown and TRAIP-overexpressing groups; histograms showing the percentage of Ki67-positive cells. （E）Flow cytometry showing the cell cycle distribution of the cell lines (*** p < 0.001, ** p < 0.01, * p < 0.05). （F）Wound healing assays were used to determine the effects of TRAIP knockdown or overexpression on cell migration. （G）Trans well assay showing the number of cells migrating in the TRAIP knockdown or overexpression group.
