# Supplementary material for: TRAIP promotes the development of papillary thyroid cancer by inhibiting TRAF2-mediated BRAF ubiquitination
Source: J Biol Chem. 2026 Jun 12;302(8):113246. doi: 10.1016/j.jbc.2026.113246 (PMC13355724; doi:10.1016/j.jbc.2026.113246)
Supplement: Supporting information 3 [file mmc5.docx]

**Sequence of the TRAIP mutant.**

The regions highlighted in red denote the locations of genetic mutations.

atgcctatc

121 cgtgctctgG CCactatcG CCtccgacttc ttcgatcact cccgcgacgt ggccgccatc

181 cactgcggcc acaccttcca cttgcagtgc ctaattcagt ggtttgagac agcaccaagt

241 cggacctgcc cacagtgccg aatccaggtt ggcaaaagaa ccattatcaa taagctcttc

301 tttgatcttg cccaggagga ggagaatgtc ttggatgcag aattcttaaa gaatgaactg

361 gacaatgtca gagcccagct ttcccagaaa gacaaggaga aacgagacag ccaggtcatc

421 atcgacactc tgcgggatac gctggaagaa cgcaatgcta ctgtggtatc tctgcagcag

481 gccttgggca aggccgagat gctgtgctcc acactgaaaa agcagatgaa gtacttagag

541 cagcagcagg atgagaccaa acaagcacaa gaggaggccc gccggctcag gagcaagatg

601 aagaccatgg agcagattga gcttctactc cagagccagc gccctgaggt ggaggagatg

661 atccgagaca tgggtgtggg acagtcagcg gtggaacagc tggctgtgta ctgtgtgtct

721 ctcaagaaag agtacgagaa tctaaaagag gcacggaagg cctcagggga ggtggctgac

781 aagctgagga aggatttgtt ttcctccaga agcaagttgc agacagtcta ctctgaattg

841 gatcaggcca agttagaact gaagtcagcc cagaaggact tacagagtgc tgacaaggaa

901 atcatgagcc tgaaaaagaa gctaacgatg ctgcaggaaa ccttgaacct gccaccagtg

961 gccagtgaga ctgtcgaccg cctggtttta gagagcccag cccctgtgga ggtgaatctg

1021 aagctccgcc ggccatcctt ccgtgatgat attgatctca atgctacctt tgatgtggat

1081 actcccccag cccggccctc cagctcccag catggttact acgaaaaact ttgcctagag

1141 aagtcacact ccccaattca ggatgtcccc aagaagatat gcaaaggccc caggaaggag

1201 tcccagctct cactgggtgg ccagagctgt gcaggagagc cagatgagga actggttggt

1261 gccttcccta tttttgtccg gaatgccatc ctaggccaga aacagcccaa gaggcccagg

1321 tcagagtcct cttgcagcaa agatgtggta aggacaggct tcgatgggct cggtggccgg

1381 acaaaattca tccagcctac tgacacagtc atgatccgcc cattgcctgt taagcccaag

1441 accaaggtta agcagagggt gagggtgaag acagtgcctt ctctcttcca ggccaagctg

1501 gacaccttcc tgtggtcgtg a
